# *MiR-495-3p* regulates cell migration and invasion in papillary thyroid carcinoma

**DOI:** 10.3389/fonc.2023.1039654

**Published:** 2023-01-26

**Authors:** Letícia Ferreira Alves, Murilo Vieira Geraldo

**Affiliations:** Department of Structural and Functional Biology, University of Campinas (UNICAMP), São Paulo, Brazil

**Keywords:** *miR-495-3p*, papillary thyroid carcinoma, cell migration, cell invasion, bioinformatics

## Abstract

**Background:**

Papillary thyroid carcinoma (PTC) is the most prevalent histotype of thyroid cancer and the presence of BRAFV600E mutation in these tumors is related to the malignancy and prognosis of the disease. In recent years attention has been focused on the role of microRNAs in the biology of PTC cells, especially in their role in the modulation of pathways related to tumorigenesis. DLK1-DIO3-derived miRNAs have been shown to play important roles in tumor context and are globally downregulated in PTC.

**Methods:**

Based on a previous in silico target prediction and gene enrichment analysis, we identified miR-495-3p as the candidate with the highest tumor suppressor potential role in PTC among DLK1-DIO3-derived miRNAs. We used bioinformatics and an in vitro model of miR-495-3p overexpression to further understand the influence of this molecule on the tumorigenic processes of PTC.

**Results:**

Overexpression of miR-495-3p impaired cell migration and invasion of PTC cells harboring the BRAFV600E mutation and affected the expression of targets predicted in the bioinformatic analysis, such as TGFB2, EREG and CCND1.

**Conclusion:**

Overall, our results indicate that the loss of miR-495-3p expression during PTC development might play an important role in its progression.

## 1 Introduction

Carcinoma is the prevalent form in which malignant tumors are observed in the thyroid gland. The group of well-differentiated carcinomas includes the follicular subtype (FTC), which represents about 15% of cases, and the papillary subtype (PTC), which represents about 85% of all cases of thyroid cancer in the US (1). The best characterized genetic alteration in PTC is the one that encodes the BRAF^V600E^ oncoprotein. This mutation is the most common genetic alteration found in PTC and is associated with more aggressive biological properties of papillary carcinoma (2–4). In the last decade, the number of studies exploring the abnormal expression of miRNAs as molecular markers for cancer diagnosis and prognosis increased in the literature (5–8). Regarding the biological role of miRNAs, their interaction with mRNAs constitutes an overly complex network, especially in humans. A single miRNA regulates a miscellaneous of mRNAs, and a single gene might be under the control of a wide variety of miRNAs, generating a robust network of post-transcriptional regulation involved in multiple cell processes, such as cell differentiation, metabolic regulation, and apoptosis (9).

Studies on distinct types of cancer show correlation between tumorigenesis and miRNA roles (10–13). Moreover, diverse miRNAs present tumor promoter or suppressor roles, indicating the intricate function of these molecules in the development and progression of thyroid neoplasms (14–16).

The aberrant levels of miRNAs found in thyroid tumor samples and the relationship of these molecules with classic oncogenes and tumor suppressors highlight this subtype of RNAs as important therapeutic targets. The long arm of chromosome 14 hosts the largest miRNA cluster in the human genome, known as DLK1-DIO3 region. This region is highly conserved and harbors more than 50 miRNA genes (17). The large-scale analysis of miRNA expression in a PTC murine model revealed the global downregulation of several miRNAs situated in the DLK1-DIO3 genomic region (18). Altogether, the comprehension of the functional role of these molecules in thyroid cells offers tumorigenesis intervention perspectives and remains unclear.

The intricate and complex post-transcriptional regulation network which miRNAs determine is the principal limitation for functional analysis involving these molecules. To overcome this limitation, we previously performed the bioinformatic prediction of the potential regulation network controlled by DLK1-DIO3 region miRNAs (19). The results pointed *miR-495-3p* as one of the top-ranked miRNAs from DLK1-DIO3 region regarding the number of targets involved with several cell processes. Importantly, when only targets involved with cancer-related processes or oncogenes were analyzed, *miR-495-3p* stood out in the first position of the ranking.

The literature indicates involvement of *miR-495-3p* with the suppression of several types of tumors (e.g., mammary, prostate, gastric and glioma), by targeting key factors for carcinogenesis (20–23). As *miR-495-3p* emerges as a promising candidate in the study of thyroid oncogenesis and progression, this study aimed to investigate the biological role of *miR-495-3p* in PTC development and progression.

## 2 Material and methods

### 2.1 *miR-495-3p* target prediction

MiRWalk version 2.0 (24) was used to predict miRNA-target sequence-based interactions by 12 different algorithms. Only interactions predicted by TargetScan and 6 more algorithms were considered valid. The resulting list was filtered to only keep genes that were found upregulated in our Differential Gene Expression analysis. The construction of a Protein-Protein Interaction (PPI) network and STRING enrichment analysis of predicted targets were performed using Cytoscape 2.0 (25).

### 2.2 Differential expression analysis

To investigate PTC’s expression landscape, we downloaded 570 RNA-seq datasets available for normal and tumor thyroid samples on GDC Data Portal (TCGA-THCA project, downloaded on August 4^th^, 2020). R programming language (version 4.0.4) was used for data manipulation and analysis. We started our analysis by joining all the data on a single file, samples that were of no use for our analysis were excluded. The exclusion factors were: non-PTC tumor samples; non-primary (metastatic) samples; non-BRAF^V600E^ point mutations; samples with non-available metadata and with unknown BRAF status. By the end of sample trimming the resulting 536 samples were split into 58 normal (healthy tissue) samples and 478 primary tumor samples.

With data filtered and joined, quality control and differential expression analysis were performed using the DESeq2 Bioconductor package (26). Genes were considered differentially expressed when abs(log2FC) > 0.58 and P-adjusted value <0.01. Category netplot was created using the DOSE Bioconductor package (27), where the top five enriched categories among DEG genes are shown.

miRNA-seq files were also downloaded from GDC Data Portal as described above. Given the low levels and wide variation of miRNAs expression, only paired samples from our dataset were used to analyze *miR-495-3p* expression. 55 pairs of samples were kept after filtering. Fold changes were calculated after normalization of counts.

### 2.3 Weighted gene coexpression network analysis

We used WGCNA to identify coexpressed genes in the list of differentially expressed genes (DEG) generated as described above. WGCNA analysis was performed following procedures indicated by the package developers (28). Soft thresholding was set to 5. The genes in the modules obtained were crossed with the list of *miR-495-3p* predicted targets to find target representation for each module. GO enrichment analyses were performed for the modules with target representation >10% and composed of more than 80 genes.

### 2.4 Cell culture

N-Thy-ORI, TPC-1, BCPAP and KTC cell lines were a courtesy of Professor Edna Teruko Kimura (University of Sao Paulo, Brazil). Cell lines were cultivated in conditions described in **Table 1**.

**Table 1:** Cell line features and culture conditions.

Cell line	Features	Genetic alterations	Media	FBS	Supplementation
TPC-1	Derived from PTC	RET/PTC1 (spontaneous)	DMEM	5%	–
BCPAP	Derived from PTC	*BRAF^V600E^* (spontaneous)	DMEM	10%	–
KTC-2	Derived from ATC	*BRAF^V600E^* (spontaneous)	RPMI	5%	–
N-Thy-ORI	Normal immortalized	–	RPMI	10%	2 mM L-glutamine

All experimental groups were maintained in a 37°C incubator with 5% of CO_2_ with antibiotics (penicillin 100 U/mL and streptomycin 100 μg/mL, Thermo Fisher) and antifungal (amphotericin B 1 μg/mL, Thermo Fisher).

### 2.5 DNA constructs and plasmid transfection

The *MIR495* genomic region was amplified and cloned in pGEM-T Easy Vector System (Promega). Then, the insert was removed from the plasmid by double digestion with *Xho*I and *Eco*RI and ligated in MSCV puro vector previously digested with the cited restriction enzymes. The presence and integrity of the insert was confirmed by PCR amplification and sanger sequencing. Transfection of the plasmid constructions were performed using Lipofectamine 2000 (Thermo Fisher) according to manufacturer’s instructions. Following transfection, the cell lines were maintained in cell medium containing 5 μg/mL of puromycin. Overexpression of *miR-495-3p* was confirmed by RT- qPCR.

### 2.6 Scratch assay

Forty-to-sixty thousand cells were plated in a 24 well plate, in triplicates. Following transfection, a wound was made by scratching the plate with a pipette tip. Photomicrographs were taken with a Nikon Eclipse E600 microscope (40x magnification), after 16 and 24 h of the scratch making and the quantitative analysis was performed with ImageJ by measuring the wound’s area of each acquired field and comparing the results statistically.

### 2.7 Transwell assay

The cell migration and invasion assays were performed using 8.0 μm pore membrane inserts (Millipore, MA). Twenty thousand cells were resuspended in cell media containing 0.5% fetal bovine serum and plated in the upper chamber compartment. The lower compartment was filled with DMEM with 10% FBS. After 12 h, the cell media was removed, and the chamber was washed twice with PBS. The cells in the upper compartment were removed with cotton swabs and the cells in the lower compartment were then fixed, stained with 0.5% crystal violet, and photographed under a Nikon Eclipse E600 microscope (100x magnification). For cell invasion, 30 μL of extracellular matrix gel (ECM Gel from Engelbreth-Holm-Swarm murine sarcoma - liquid, BioReagent 8.42 μg) diluted on DMEM were plated on top of each insert and after 1h the same number of cells were seeded on top of the ECM coat.

### 2.8 Expression analysis

For total RNA extraction, 2x10^5^ cells were seeded in a 60 mm cell culture plastic dish. Cells were collected in TRIzol after 72 h. RNA extraction protocol was performed according to Chomczynski & Sacchi, (29). *miR-495-3p* and RNU6B (endogenous control) expression were analyzed using Taqman MiRNA Assays Kit (Thermo Fisher), specific for each molecule. Ten nanograms of total RNA was used for cDNA synthesis using Taqman miRNA Reverse Transcription kit (Thermo Fisher), according to the manufacturer instructions.

cDNA synthesis for *miR-495-3p* predicted targets was performed according to *Invitrogen’s M-MLV* instructions with 1 μg of total RNA per reaction. RPL19 was used as endogen control. The oligonucleotides used for qPCR reaction are shown in **Supplementary Table 1**. For quantification of the cDNA for miRNAs we performed the RT-qPCR reactions according to *Taqman^®^ MiRNA Assay* kit instructions, and for the targets we used the *SYBR Master Mix* (Thermo Fisher). Expression of extracellular matrix and adhesion genes was quantified using Human Extracellular Matrix & Adhesion Molecules Array plate (Thermo-Fisher), according to the manufacturer’s instructions. All amplification reactions were performed using universal cycling conditions in 7500 Real-Time PCR System (Applied Biosystems) and the differential gene expression was calculated according to Pfaffl, (30) for all reactions apart from the array plates were analyzed using ThermoCloud platform (Thermo Fisher).

### 2.9 Statistical analysis

All statistical analyses were performed using GraphPad Prism (version 5.0). Graphically, results are presented as mean ± standard errors of the means (SEMs). Functional and gene expression data were submitted to Student’s t test for comparisons between two groups and statistical significance was considered when P<0.05.

## 3 Results

### 3.1 *miR-495-3p* is downregulated in PTC

**Figure 1:**
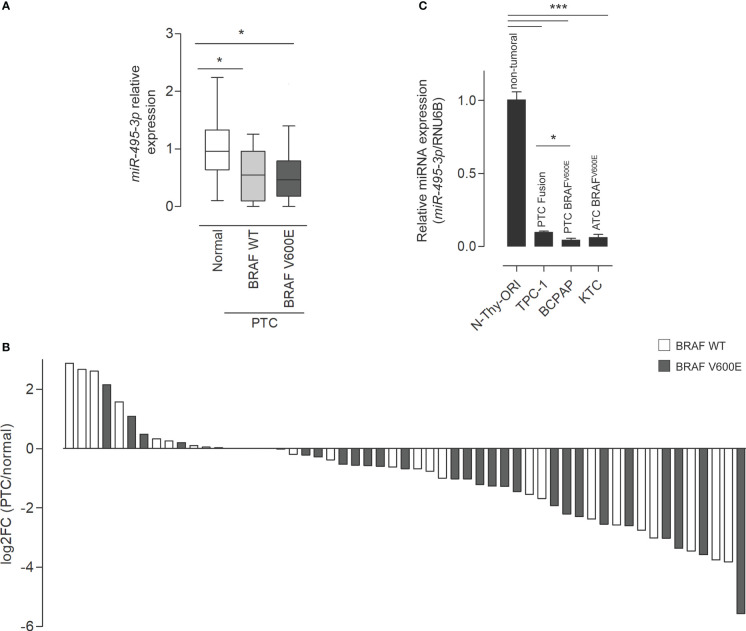
*miR-495-3p* is down regulated in PTC. **(A)** Boxplot (Whiskers: Tukey) shows the differential expression between normal samples and tumor samples bearing or not the BRAF^V600E^ mutation (individual normalized RPM values/mean of normalized RPM values for the normal group). **(B)** Waterfall plot for 55 paired tumor/healthy tissue samples analyzing *miR-495-3p* expression shows that the downregulation of *miR-495-3p* is a common event during carcinogenesis. Expression data for the plot is from TCGA. Gray bars on the waterfall plot represent BRAF^V600E^-positive samples and white bars the PTC samples where the mutation is absent. **(C)** Bar plot shows the expression of *miR-495-3p* in four different thyroid cell lines. As previously described, the malignant cells have progressive decreases in *miR-495-3p* expression. Data are presented as the average of triplicates of a single experiment and bars represent the standard errors of the means (SEMs). *P<0.05, ***P<0.001.

The underexpression of *miR-495-3p* in PTC was previously identified by our group both in human samples and in the transgenic mouse model Tg-Braf (18). Additionally, a computational analysis pointed *miR-495-3p* as the key modulator of cancer-related genes in thyroid cancer datasets (Marson & Alves et al.; in review). The analysis of TCGA expression data from 55 paired samples revealed that *miR-495-3p* is underexpressed in tumor context (**Figure 1A**). In this cohort, we observed downregulation of *miR-495-3p*, with no significant impact of BRAF^V600E^mutation on the expression of *miR-495-3p* in PTC samples (**Figures 1A, B**). Moreover, *miR-495-3p* is downregulated in the three thyroid cancer cell lines analyzed (TPC-1, BCPAP, KTC) in comparison with the non-tumoral N-Thy-ORI cells, with lower levels observed in the cell lines with a more aggressive phenotype (**Figure 1C**). Interestingly, the expression of *miR-495-3p* is significantly lower in the *BRAF^V600E^*-positive cell lines (BCPAP and KTC-2) when compared to the tumoral *BRAF^V600E^-negative* cell line (TPC-1).

### 3.2 Post-transcriptional regulation by *miR-495-3p* in PTC

**Figure 2:**
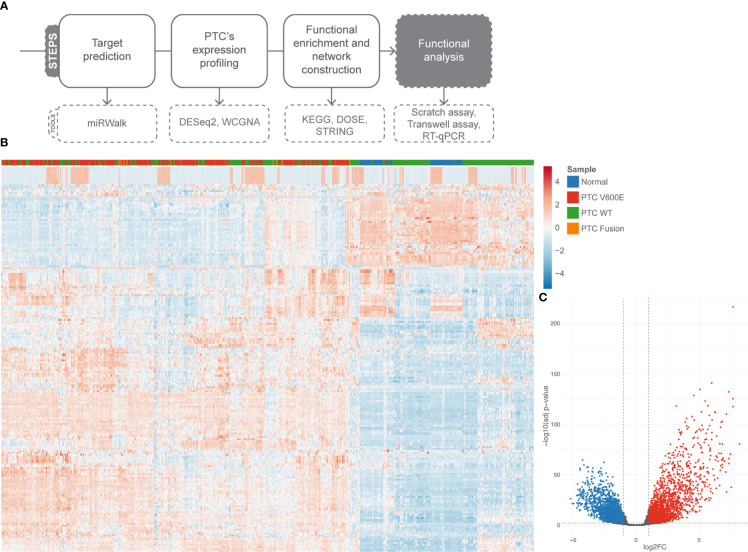
Differentially expressed genes in PTC. **(A)** Pipeline of investigation shows, briefly, the steps we followed to validate *miR-495-3p* influence on PTC progression. **(B)** Heatmap of the top 200 DEG (abs(log2FC) > 0.58 and adjusted p value < 0.01). **(C)** Volcano plot of DGE analysis. Upregulated genes are shown in red and down regulated genes in blue.

**Figure 3:**
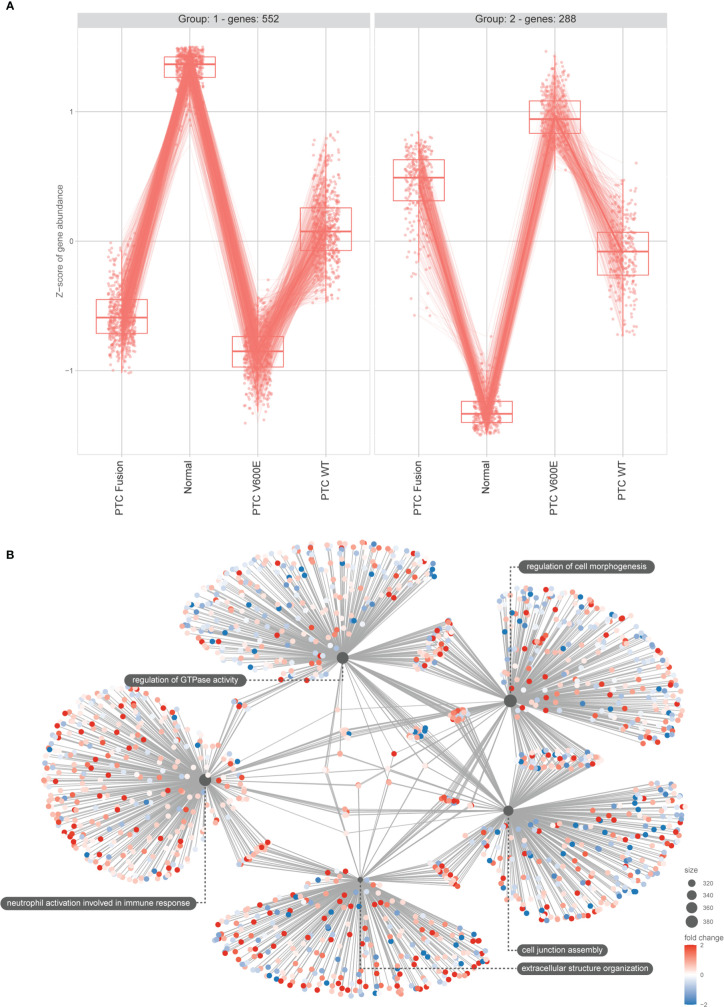
DEGs main clusters reinforce the BRAF^V600E^ signature on PTC samples and functional enrichment reveals top modulated processes. **(A)** The plot shows the result of the clustering of the top 1000 DEGs obtained using LRT for the mRNA seq dataset. Min genes = 100. **(B)** Category netplot shows the correlation between the genes associated with the top five most enriched GO terms and the fold changes of the significant genes associated with these terms (colors).

The pipeline used from bioinformatic investigation to functional validation is illustrated on **Figure 2A**. To take a better view on PTC’s gene expression landscape we performed differential gene expression (DGE) analysis of 536 samples (58 healthy tissue samples and 478 tumor samples) (**Figures 2B, C**). To assess the impact of *BRAF* mutation status on the post-transcriptional regulation by *miR-495-3p* in the PTC samples, we used the likelihood-ratio test (LRT) to test for any differences across the grouping variable. Following LRT, we used a clustering tool to group the differentially expressed genes (DEGs) based on the changes on their expression across the sample groups. Clusterization of LRT-derived DEGs reinforced the distinction of *BRAF^V600E^* and *BRAF WT* PTC samples regarding their expression levels (**Figure 3A**). Focusing on the two largest clusters (>100 genes), we observed that the *BRAF^V600E^* PTC samples show higher differences from the healthy tissue. Next, we used the pairwise approach to look for DEGs between healthy tissue (normal) samples and PTC *BRAF^V600E^* samples. Category netplot of these DEGs revealed the top 5 enriched categories among these genes: cell junction assembly, regulation of cell morphogenesis, extracellular matrix organization, extracellular structure organization and regulation of GTPase activity (**Figure 3B**).

**Figure 4:**
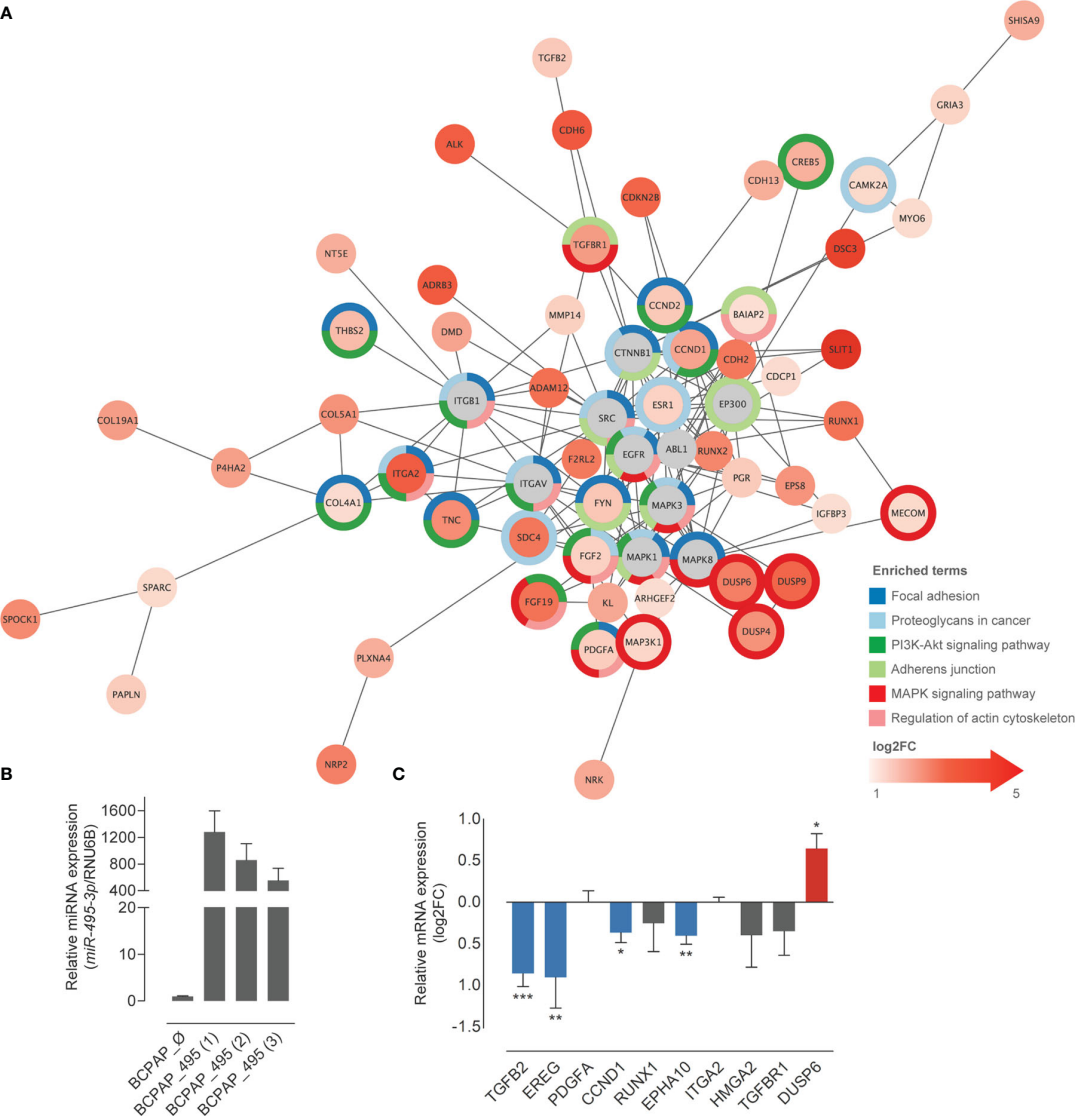
Overexpression of *miR-495-3p* causes a shift in the expression of predicted targets. **(A)** Predicted targets of *miR-495-3p* whose expression were found increased in PTC were submitted to a STRING enrichment analysis using Cytoscape. Targets with higher fold changes are shown in more intense red tones. Chart colors (borders) represent different KEGG enrichment categories. **(B)** Bar plot shows the expression of *miR-495-3p* among transfected different clones. The one with the highest expression (BCPAP_495 (1)) was used to perform functional validation. **(C)** Bar plot shows the log2FC of some of *miR-495-3p* predicted targets in BCPAP cells over expressing the miRNA. Data is presented as the average of at least three independent experiments and bars represent the standard errors of means (SEMs). *RPL19* was used as the endogen control. Student’s tests were used to compare expression of each gene between groups (*P<0.05, **P<0.01, ***P<0.001).

Based on the observations above and in the support of literature on the relevance of the *BRAF^V600E^* mutation to malignancy and prognosis of PTC, we decided to further explore the influence of *miR-495-3-p* in this condition. The list of *miR-495-3p* predicted targets was filtered to exclude downregulated DEGs on the PTC *BRAF^V600E^* context, meeting the inverse correlation expected from the miRNA-target dynamics. PPI network of the resulting filtered list revealed the potential interactions among the predicted targets. Functional enrichment of network nodes revealed processes that are essential for tumor progression, such as Focal adhesion, Proteoglycans in cancer, PI3K-Akt signaling pathway and Regulation of actin cytoskeleton (**Figure 4A**).

Ten genes from the list of *miR-495-3p* targets were selected for experimental validation. We generated BCPAP (PTC BRAF^V600E^-positive cell line) stably overexpressing *miR-495-3p* (**Figure 4B**). Overexpression of *miR-495-3p* in these cells resulted in the downregulation of *TGFB2*, *CCND1*, *EPHA10* and *EREG*, corroborating our bioinformatic findings (**Figure 4C**).

### 3.3 *miR-495-3p* targets representation in WGCNA modules

**Figure 5:**
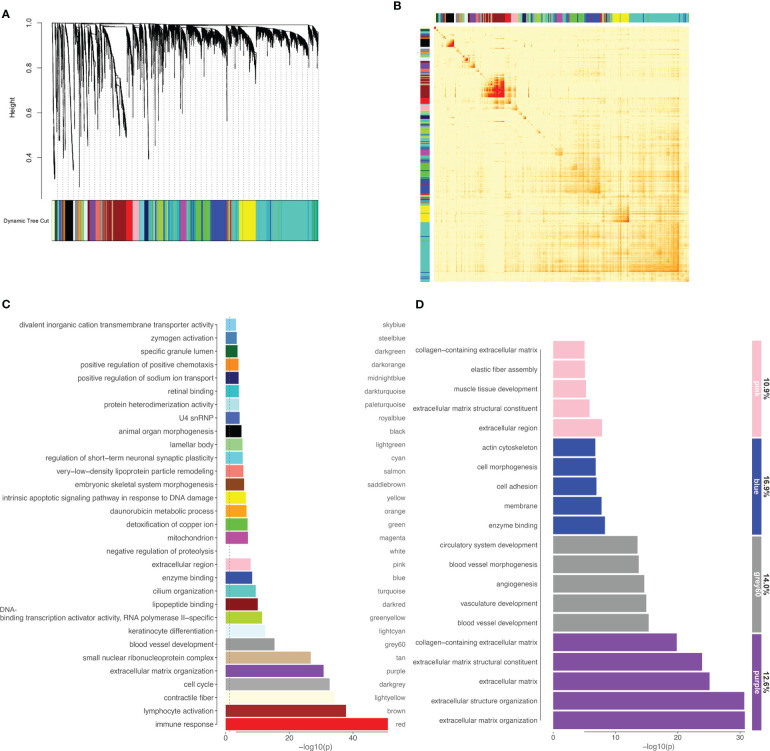
*miR-495-3p* target representation on WGCNA modules. **(A)** WGCNA dendrogram of differentially expressed genes (clustered based on 1-TOM) **(B)** Heatmap of the topological overlap matrix. Rows and columns correspond to single genes, light colors represent low topological overlap, and progressively darker orange and red colors represent higher topological overlap. **(C)** Bar plot shows the top enriched category for each module from WGCNA. **(D)** Bar plot shows GO enriched categories for WGCNA modules (>80 genes) with higher *miR-495-3p* target representation. Numbers on the right indicate the target representation for each module.

Well known for its utility on identifying coexpressed gene modules in large datasets, weighted gene coexpression network analysis (WGCNA) was used to further understand the correlation patterns among DEGs. The analysis resulted in 31 modules of coexpressed genes (**Figures 5A, B**). Functional enrichment was performed for all WGCNA modules (**Figure 5C**) and the modules were then crossed with the list of *miR-495-3p* predicted targets (**Figure 5D**). Interestingly, the modules with high target representation include enriched categories mostly related to cell adhesion (**Figure 5D**, blue), angiogenesis (**Figure 5D**, grey60) and extracellular matrix organization (**Figure 5D**, purple and pink).

### 3.4 *miR-495-3p* overexpression impairs cell migration and invasion

**Figure 6:**
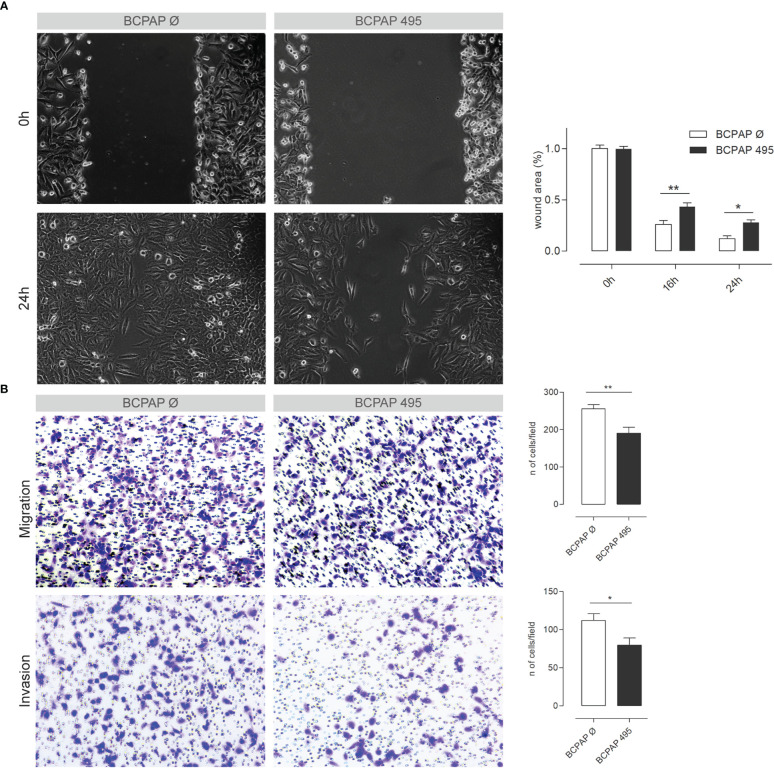
*miR-495-3p* overexpression impairs PTC cell’s migration and invasion. **(A)** Figure shows BCPAP cells stably transfected with *miR-495-3p* after 0, 16 and 24h of the wound making in the scratch assay. Cells transfected with the empty vector were used as control (40x magnification). Bar plot on the right shows the difference in wound area between the control and transfected groups after 16 and 24h of the wound making. **(B)** Representative photomicrographs of cells that migrated through the Transwell in the migration and invasion assays (100x magnification). Bar plots on the right show the count of migrated/invaded cells of both control groups and the stably transfected with *miR-495-3p*. Data is presented as one representative experiment (from three independent experiments), bars represent the standard errors of means (SEMs). Student’s t tests were used to compare values between the two groups (*P<0.05, **P<0.01).

**Figure 7:**
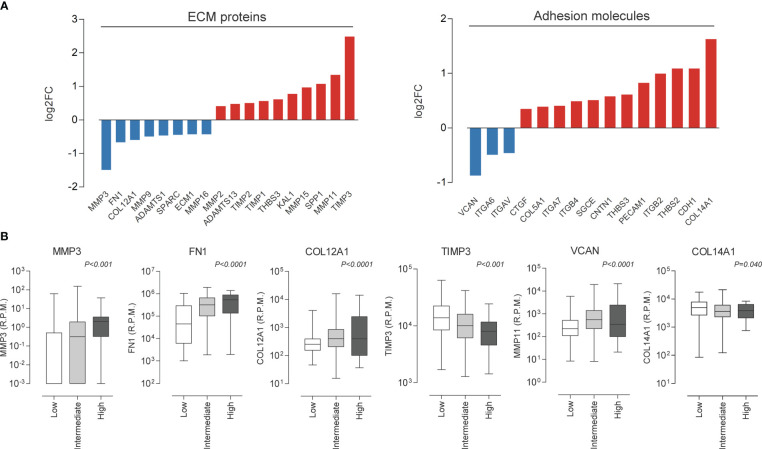
*miR-495-3p* overexpression modulates migration/adhesion related genes. **(A)** Bar plots show genes modulated by *miR-495-3p* overexpression. We considered modulated, genes from the Human Extracellular Matrix and Adhesion Molecules plate whose expression suffered a 0.25 alteration on fold-change. **(B)** Box plots show association between the expression of some genes shown on “a” and risk on PTC (TCGA data downloaded from cBioPortal).

Considering the recurrence of terms related to cell adhesion and migration, we decided to investigate the influence of *miR-495-3p* on these processes. As shown in **Figure 6**, overexpression of *miR-495-3p* significantly impaired cell migration both in the scratch and Transwell assay (**Figures 6A, B**). Cell invasion was also significantly impaired in front of *miR-495-3p* overexpression (**Figure 6B**). Importantly, the overexpression of *miR-495-3p* also induced a transcriptional reprogramming of adhesion/migration-related genes. As shown in figure 7, we observed a major impact on the expression of key genes for cell migration and invasion such as *MMP3, FN1, TIMP3* and *VCAN* (**Figure 7A**), most of them whose aberrant expression is associated with increased risk in PTC (**Figure 7B**). Finally, no significant changes were observed on the expression of genes related to thyroid cell differentiation (*TG*, *TPO*, *SLC5A5* and *TSHR*) key players on the tumor progression and survival rates in PTC (data not shown).

## 4 Discussion

Alterations in miRNAs’ expression pattern between healthy and tumor tissues have been reported in several types of cancer, establishing a link between the modulation of these molecules and tumor development and progression (31). MiRNAs with abnormal expression in cancer cells may act as oncogenes or tumor suppressors, depending on the targets they regulate. The biological function of DLK1-DIO3-derived miRNAs in PTC has been previously explored by our group, revealing decreased expression of *miR-495-3p* in PTC samples and in thyroid tumor tissue derived from transgenic mice (18). Here we show that *miR-495-3p* plays a central role in the regulation of cell migration and invasion in PTC, key processes for tumor progression, suggesting a potential tumor suppressor role for this molecule in PTC.

*MiR-495-3p*’s capacity of interacting with several key modulators of cancer-related processes (e.g., cell growth, migration, apoptosis, and angiogenesis) indicates its potential as an important regulator of tumor cells malignancy (21, 23, 32–34). The analysis of the PTC expression landscape revealed that larger shifts of gene expression are found between healthy tissue and tumor samples with the *BRAF^V600E^* mutation, confirming data from the literature. In our panel of cell lines, we observed a pattern of decreasing *miR-495-3p* expression according to the degree of differentiation of each cell line, where the cell line related to a less aggressive phenotype (TPC-1) showed levels of expression closer to the non-malignant cell line (N-Thy-ORI) whereas the more aggressive cell lines (BCPAP, KTC-2) presented lower levels of expression of the molecule.

The bioinformatic investigation of *miR-495-3p* targets revealed a myriad of genes potentially regulated by this molecule in PTC, several of them involved with crucial processes for cancer genesis and progression. We observed that a selected panel of predicted target genes were modulated in response to the *miR-495-3p* overexpression in thyroid cancer cell line. To narrow our focus and better understand how this complexity of predicted interactions could be affecting PTC development and progression, we crossed the data obtained for *miR-495-3p* predicted targets with the analysis of PTC expression landscape. The results showed the involvement of this miRNA in highly enriched processes in PTC (e. g. Focal adhesion, Proteoglycans in cancer, PI3K-Akt signaling pathway and Regulation of actin cytoskeleton). Further, we crossed the list of predicted targets with WGCNA modules of DEG to check the distribution of *miR-495-3p* predicted targets on these groups of coexpressed genes. Higher target representation was found in the modules whose enrichment revealed categories similar to the ones previously identified.

To validate the involvement of *miR-495-3p* in the most recurrent processes obtained in our bioinformatics investigation, we performed functional assays and observed that the overexpression of this miRNA alters the pattern of migration and invasion of BCPAP cells. These results corroborate previous studies concerning the biological function of the whole DLK1-DIO3 region which revealed the involvement of the miRNAs from this region in the regulation of focal adhesion and extracellular matrix remodeling, essential processes for cell migration (35–37). Further, we have shown that the overexpression of this single miRNA resulted in the reprogramming of important genes for cell migration, adhesion, and extracellular matrix remodeling. Some of the modulated genes, such as *MMP3, FN1, COL12A1* and *VCAN* have their expression associated with high risk and moderate extrathyroidal invasion in PTC samples, according to TCGA data. We believe that the modulations on gene expression are consistent with those observed in the functional assays and reinforce the influence of *miR-495-3p* on the above-mentioned processes. Overall, our results reveal *miR-495-3p* as a promising tumor suppressor which plays a role in the regulation of key processes on genesis and progression of PTC.

## Data availability statement

The original contributions presented in the study are included in the article/**Supplementary Material**. Further inquiries can be directed to the corresponding author.

## Author contributions

Experiments were planned and designed by both authors. LA carried out all bioinformatic and functional analysis. Manuscript was written and revised by both authors. All authors contributed to the article and approved the submitted version.
