# Supplementary material for: MiR-495-3p regulates cell migration and invasion in papillary thyroid carcinoma
Source: Front Oncol. 2023 Jan 26;13:1039654. doi: 10.3389/fonc.2023.1039654 (PMC9911110; doi:10.3389/fonc.2023.1039654)
Supplement: Supplementary file 1 [file Table_1.pdf]

Gene	Forward primer	Reverse primer
TGFB2	TTCAGACACTCAGCACAGCA	TTGGGTGTTTTGCCAATGTA
EREG	TACTGCAGGTGTGAAGTGGG	GTGGAACCGACGACTGTGAT
PDGFA	GGGAACGCACCGAGGAAG	GGAGGAGAACAGGGAGTGC
CCND1	CCTGTCCTACTACCGCCTCA	TGGGGTCCATGTTCTGCT
RUNX1	CAGATGCAGGATACAAGGCAGA	CTGGGTGCACAGAAGGAGAG
EPHA10	CGCTACAAGGACAGCTTCG	TCGATGTTCAGCCAAAGAGA
ITGA2	GCTGGAATCCTTTTGCTGTT	GCAGGTAGGTCTGCTGGTTC
HMGA2	ACTTCAGCCCAGGGACAAC	CTTGTTTTTGCTGCCTTTGG
TGFBR1	AAATTGCTCGACGATGTTCC	TTCAACTGATGGGTCAGAAGG
DUSP6	CTAGACGGCTCGTGTAGCAG	CTCGGTCAAGGTCAGACTCG
